# Porcine Epidemic Diarrhea Virus nsp15 Antagonizes Interferon Signaling by RNA Degradation of TBK1 and IRF3

**DOI:** 10.3390/v12060599

**Published:** 2020-05-31

**Authors:** Yang Wu, Hongling Zhang, Zhaorong Shi, Jianfei Chen, Mingwei Li, Hongyan Shi, Da Shi, Longjun Guo, Li Feng

**Affiliations:** 1 State Key Laboratory of Veterinary Biotechnology, Harbin Veterinary Research Institute, Chinese Academy of Agricultural Sciences, Harbin 150069, China; wuyang_neau@126.com (Y.W.); ha163zhl@163.com (H.Z.); szr20201229@126.com (Z.S.); chenjianfei@caas.cn (J.C.); limingwei101825@163.com (M.L.); shihongyan@caas.cn (H.S.); shida@caas.cn (D.S.); 2 College of Animal Science and Veterinary Medicine, Heilongjiang Bayi Agricultural University, Daqing 163319, China

**Keywords:** PEDV, TBK1, IRF3, RNA degradation, interferon signaling

## Abstract

Porcine epidemic diarrhea virus (PEDV) causes a porcine disease associated with swine epidemic diarrhea. The type I interferon (IFN-I or IFN α/β) is a key mediator of innate antiviral response during virus infection. Different antagonistic strategies have been identified and determined as to how PEDV infection inhibits the host’s IFN responses to escape the host innate immune pathway, but the pathogenic mechanisms of PEDV infection are not fully elucidated. Our preliminary results revealed that endogenous TANK-binding kinase 1 (TBK1) and interferon regulatory factor 3 (IRF3), the key components in the IFN signaling pathway were downregulated in PEDV infected IPEC-J2 cells by iTRAQ analysis. In this study, we screened nsp15 as the most important viral encoded protein involved in TBK1 and IRF3 reduction. Endoribonuclease (EndoU) activity has been well determined for coronavirus nsp15. Three residues (H226, H241, and K282) of PEDV nsp15 were identified as critical amino acids for PEDV EndoU but not D265, which was not well correlated with published results of other coronaviruses, such as severe acute respiratory syndrome virus (SARS-CoV). Moreover, PEDV nsp15 can directly degrade the RNA levels of TBK1 and IRF3 dependent on its EndoU activity to suppress IFN production and constrain the induction of IFN stimulated genes (ISGs), by which PEDV antagonizes the host innate response to facilitate its replication. Collectively, these results have confirmed that PEDV nsp15 was capable of subverting the IFN response by the RNA degradation of TBK1 and IRF3.

## 1. Introduction

Porcine epidemic diarrhea (PED) is caused by porcine epidemic diarrhea virus (PEDV), which has a positive-strand RNA genome of 28 kb in length in the genus *Alphacoronavirus*, family Coronaviridae and order Nidovirales [1,2,3]. The disease is characterized by severe enteritis, vomiting, watery diarrhea, dehydration, and a high mortality rate among swine [4]. Two-thirds of the genome (ORF1a and 1b) encode a large replicase polyprotein, whereas the remainder of the genome encodes for structural proteins and accessory proteins [5,6]. After PEDV virus entry into the host cells, ORF1a codes for a large polyprotein 1a (pp1a), while ORF1b is expressed as the pp1ab fusion protein via the ribosomal frameshifting. By the proteinase activity of nsp3 (a papain-like protease) and nsp5 (a main protease), these polyproteins are proteolytically processed to 16 nonstructural proteins (nsp1 to 16), which mediate the replication of the viral RNA genome and synthesis of a nested set of subgenomic mRNAs [6]. In late 2010, a newly emerging PEDV variant was reported with more virulence and higher mortality in suckling piglets, compared to the classical PEDV that was first discovered in Europe in 1971 [7]. To date, the newly emerging PEDV variant is recognized as the major infectious pathogen for swine diarrhea-associated diseases in swine-raising farms in China and it has spread to other countries worldwide, causing a high number of pig deaths and significant economic impacts [8,9,10,11,12].

During viral infection, the innate immune response is activated, leading to the induction of the type I interferon (IFN-I or IFN α/β). IFN-I is the potent cytokine of critical importance in controlling viral infections and priming adaptive immune responses [13]. Following production, IFN-I initiates a positive feed-back loop by binding to their cognate receptors on the cell surface in an autocrine and paracrine manner [14,15] and activating JAK protein tyrosine kinases (JAK1 and Tyk2) which phosphorylate signal transducers and activators of transcription STAT1 and STAT2. STAT1 and STAT2 together with interferon regulatory factor 9 (IRF9) form a transcription factor complex termed IFN-stimulated gene factor 3 (ISGF3). Then, ISGF3 is translocated into the nucleus and binds to the IFN-stimulated response elements (ISRE) to induce the expression of IFN-stimulated genes (ISGs), which establish an antiviral state [15,16].

However, many viruses, including coronaviruses, have evolved mechanisms to evade the host immune system [17,18,19,20,21,22,23,24]. Previous studies suggest that PEDV can restrain host innate immune response by different strategies, such as by degradation or cleavage of key factors essential the IFN signaling pathway [25,26], competitive interaction between viral encoded proteins and modulators for IFN production [27,28], or localization changes of antiviral components [29]. Whether there are other mechanisms utilized by PEDV to circumvent the host response remains unclear. Our previous results have demonstrated that differentially expressed proteins were identified in PEDV infected IPEC-J2 cells by the analysis of isobaric tags for relative and absolute quantitation (iTRAQ). We identified 49 differentially expressed cellular proteins, of which eight were upregulated and 41 downregulated. These differentially expressed proteins were involved in apoptosis, signal transduction, and stress responses. In our analysis, TBK1 and IRF3, two important modulators in the activation of the interferon signaling pathway were downregulated post PEDV infection. In this study, we screened the PEDV viral proteins involved in the reduction of TBK1 and IRF3 expression. Among the identified viral proteins, nsp15 was recognized as the most effective viral protein contributing to the reduction of both TBK1 and IRF3 expression after the co-transfection of TBK1 or IRF3 with individually encoded PEDV proteins in vitro. It was confirmed that the two major well-known degradation systems, namely the ubiquitin-proteasome system or autophagy, were not involved in PEDV nsp15 mediated reduction of TBK1 and IRF3. In contrast, PEDV nsp15 was capable of suppressing TBK1 and IRF3 expression by endoribonuclease-dependent degradation of TBK1 and IRF3 RNA. This resulted in a decrease of IFN and ISG production, resulting in PEDV host innate immune escape.

## 2. Materials and Methods

### 2.1. Cell Culture and Viruses

IPEC-J2 cells (porcine small intestine epithelial cell clone J2; ATCC), Vero E6 (African green monkey kidney cell line; ATCC), and HEK293 cells (human embryonic kidney epithelial cells; ATCC) were cultured in Dulbecco’s minimum essential medium (DMEM) (Life Technologies, USA) supplemented with 10% heat-inactivated fetal bovine serum (FBS) (Gibco, USA), 100 U/mL penicillin, 100 μg/mL streptomycin at 37 °C in an incubator with 5% CO2 (Thermo Scientific, USA). PEDV strain CV777 (GenBank accession number KT323979) was prepared and titrated as previously described [30]. Vesicular stomatitis virus that expresses the green fluorescence protein (VSV-GFP) was preserved in Harbin Veterinary Research Institute, Harbin, and stored at −80 °C.

### 2.2. Plasmids and Antibodies

The full-length sequence of TBK1 and IRF3 were constructed into the pCAGGS-HA vector to obtain recombinant plasmids, pCAGGS/HA-TBK1 and pCAGGS/HA-IRF3, respectively. The recombinant pCAGGS plasmids containing individual PEDV viral protein (nsp1-10, nsp12-16, S, E, M, and N) with a Flag fusion tag were kindly provided by Prof. Yue Wang from Harbin Veterinary Research Institute. Mutagenesis of the PEDV nsp15 constructs (H226A, H241A, D265A and K282A) were performed by using site-directed mutagenesis kit (TakaRa, China). Recombinant prokaryotic expression plasmids were obtained following cloning of the individual gene of PEDV nsp15 and its mutant into the *Eco*R I and *Xho* I sites of pGEX-6P-1 plasmid vector (GE Healthcare Life Sciences). Recombinant pGEM-T/TBK1 and pGEM-T/IRF3 plasmids were generated to serve as DNA templates for an RNA transcription assay in vitro by amplifying the full length sequence of TBK1 or IRF3 into pGEM-T easy vector by the T-A ligation method. The specific primers used for the construction of target plasmids are listed in Table 1 and all the constructed plasmids were verified by DNA sequencing. The listed antibodies were used in this study including TBK1 rabbit monoclonal antibody (mAb) (Cell Signaling Technology), IRF3 rabbit mAb (Cell Signaling Technology), and phospho-IRF3 (Ser396) (4D4G), rabbit mAb (Cell Signaling Technology), anti-FLAG mouse mAb (Sigma), anti-HA mouse mAb (Sigma), IRDye-conjugated secondary antibody (Li-Cor Biosciences), and β-actin mouse mAb (Sigma).

### 2.3. Virus Infection and Drug Treatments

Monolayers of Vero E6 and IPEC-J2 cells were infected with PEDV strain CV777 at multiplicity of infection (MOI) of 0.1 for 1 h at 37 °C. Unbound virus was removed, and cells were maintained in complete medium for various time points until samples had been harvested. Some cell samples were treated with proteasome inhibitor MG132 (Sigma) at the concentration of 2 µM, autophagy inhibitor 3-Methyladenine (3-MA, Sigma) at 5 mM, or carrier control DMSO during some transfection assays as previously described [25].

### 2.4. Transfection

HEK293 cells were transfected with indicated plasmids using X-tremeGENE transfection reagent according to manufacturer’s instruction (Roche, USA). At 36 h post transfection, cell samples were collected and lysed in RIPA buffer (Beyotime, Nantong, China) for the Western blot analysis of targeted proteins.

### 2.5. IFA

Immunofluorescence assays (IFA) were performed as described previously with slight modification [25]. Briefly, HEK293 cells were co-transfected with TBK1, IRF3 together with nsp15, nsp15 mutants, or empty vector control followed by the collection of supernatants for each treatment at 30 h post transfection. IPEC-J2 cells were treated with the collected supernatants with three-fold dilution for 12 h followed by inoculation of VSV-GFP at MOI of 0.1. The fluorescence was visualized at 10 hpi with an Olympus inverted fluorescence microscope equipped with a camera.

### 2.6. Western Blot

Western blot analysis was performed as previously described with a slight modification [31]. Treated samples were lysed in radioimmunoprecipitation assay (RIPA) buffer (HaiGene, China) containing protease inhibitor cocktail and phosphatase inhibitors (Roche, Switzerland), separated by SDS-PAGE under reducing conditions, and transferred onto a PVDF membrane (Merck Millipore, USA). After blocking, the membranes were incubated with a primary antibody and then incubated with an appropriate IRDye-conjugated secondary antibody (Li-Cor Biosciences, Lincoln, NE). The membranes were scanned using an Odyssey instrument (Li-Cor Biosciences) according to the manufacturer’s instructions.

### 2.7. RNA Transcription In Vitro

Linearized DNA was prepared by digestion with restriction endonuclease *sal* I prior to in vitro transcription to produce RNA of defined length. In vitro transcribed RNA of TBK1 and IRF3 were generated from recombinant pGEM-T/TBK1 or pGEM-T/IRF3 plasmid as template, respectively, using the RiboMAX™ large scale RNA production systems (Promega, USA). Transcribed RNA was purified by removal of the DNA template and proteases following transcription reaction as the manufacturer’s instruction (Promega, USA) and stored for nuclease assay at –80 °C.

### 2.8. Protein Expression and Purification

For protein expression, individual plasmid of pGEX-6P-1-PEDV nsp15, pGEX-6P-1-PEDV nsp15 mutant derivatives (H226A, H241A, D265A, and K282A), or pGEX-6P-1 empty vector was transformed to Escherichia coli BL21 (DE3) cells, respectively. The Glutathione S-transferase (GST) fusion proteins were expressed following isopropyl-β-D-thiogalactopyranoside (IPTG) inductions and purified by affinity chromatography using glutathione immobilized to a sepharose matrix per the manufacturer’s instruction (GE Healthcare Life Sciences, USA).

### 2.9. Endoribonuclease Assay

The endoribonuclease activity assay was done as previously described [32]. Briefly, nuclease reactions contained 4 μg of purified wild-type PEDV nsp15 protein, PEDV nsp15 mutant protein, or GST tag protein as control, and 6 μg TBK1 or IRF3 RNA transcribed and purified in vitro. Reactions were performed in 25 mM Hepes-KOH (pH 7.4)/50 mM NaCl/5 mM MnCl_2_/1 mM DTT. Following incubation at 37 °C for 1 h, the reactions were extracted using phenol-chloroform-isoamyl alcohol and analyzed by agarose-formaldehyde gel electrophoresis.

### 2.10. Northern Blot

For northern blot, total RNA was harvested by using Trizol reagent (Invitrogen, USA) and analyzed by agarose-formaldehyde gel electrophoresis. RNAs were transferred to a 0.45-μm nylon membrane and probed with biotin-labeled DNA probes generated with the specific primers (Table 1) using the North2South^TM^ biotin random prime DNA labeling kit (Thermo Scientific). The membrane was imaged an Odyssey instrument (Li-Cor Biosciences) followed by incubation with IRDye 800-conjugated streptavidin.

### 2.11. Quantitative RT-PCR

Quantitative RT-PCR analyses were carried out as described previously with a slight modification [33]. At indicated time points post transfection or PEDV infection, total RNA was extracted from cells and subjected to quantitative RT-PCR using specific primers as listed in Table 1. Relative gene quantification was performed by the 2(-Delta Delta C(T)) method [34].

### 2.12. TCID_50_ Assay

Collected virus samples were frozen and thawed three times and clarified by centrifugation at 8000× *g* for 10 min prior to titration. TCID_50_ assays were performed in Vero E6 cells following the method of Reed & Muench as previously described [34]. Briefly, cell monolayers were inoculated with serial dilutions of each virus stock and incubated for 4 days prior to observation of the presence of cytopathic effect.

### 2.13. Statistical Analysis

Variables are expressed as mean ±S.D. Data were statistically analyzed by using GraphPad Prism v5.0 software. Statistical analyses were performed using student’s *t* test. A *p* value of <0.05 was considered significant.

## 3. Results

### 3.1. Downregulation of Endogenous TBK1 and IRF3 Post PEDV Infection

**Figure 1:**
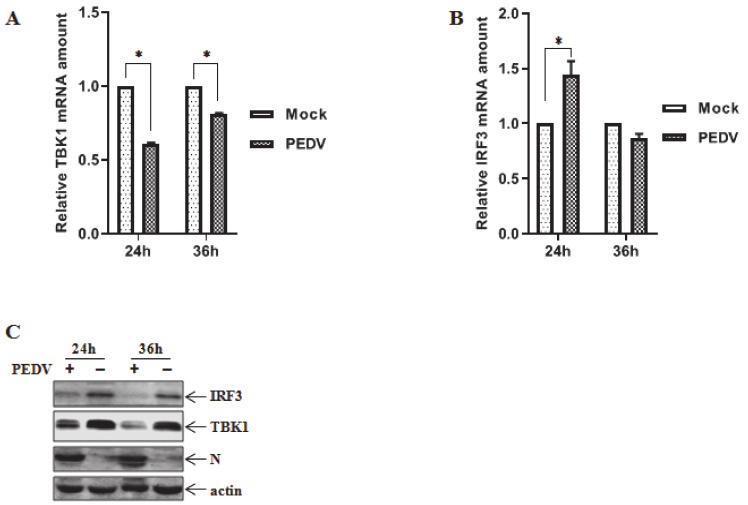
PEDV infection reduces TBK1 and IRF3 expression. (**A**,**B**) IPEC-J2 cells were infected with PEDV at MOI of 1.0 for 24 h and 36 h. Total RNA was extracted from cell samples and TBK1 and IRF3 mRNA levels were assessed by quantitative RT-PCR using the primers listed in Table 1. Three independent experiments were performed in triplicate, and values are the means ± SD for all three experiments. *, *p* < 0.05. (**C**) HEK293 cells were inoculated with PEDV at MOI of 0.1 for 24 h and 36 h followed by verification of endogenous TBK1 and IRF3 proteins by western blot analysis.

IPEC-J2 cells were infected with PEDV or left untreated as control and cell samples were collected at 24 h and 36 h for iTRAQ analysis as previously described [35]. Our preliminary results revealed that endogenous TBK1 and IRF3 were downregulated at 24 h and 36 h in PEDV infected IPEC-J2 cells by iTRAQ analysis (data not shown). Subsequently, IPEC-J2 cells were infected with PEDV at MOI of 1.0 and the mRNA levels were determined by quantitative PCR. As shown in Figure 1A, TBK1 mRNA levels were significantly decreased at 24 h and 36 h post infection (hpi). In contrast, IRF3 mRNA levels were first increased at 24 hpi and then decreased at 36 hpi, indicating no obvious changes in IRF3 mRNA levels following PEDV infection (Figure 1B). To further confirm the results, HEK293 cells were inoculated with PEDV at MOI of 0.1 and cell samples were collected for endogenous TBK1 and IRF3 detection at indicated time points post infection. Consistent with the results from IPEC-J2 cells by iTRAQ, endogenous TBK1 and IRF3 were evidently reduced at 24 hpi and 36 hpi in HEK293 cells (Figure 1C). These findings suggest that a reduction of endogenous TBK1 and IRF3 may be achieved by downregulating the mRNA transcriptional levels of TBK1 and IRF3 post PEDV infection.

### 3.2. PEDV nsp15 Is the Crucial Viral Protein For Reduction of TBK1 and IRF3

**Figure 2:**
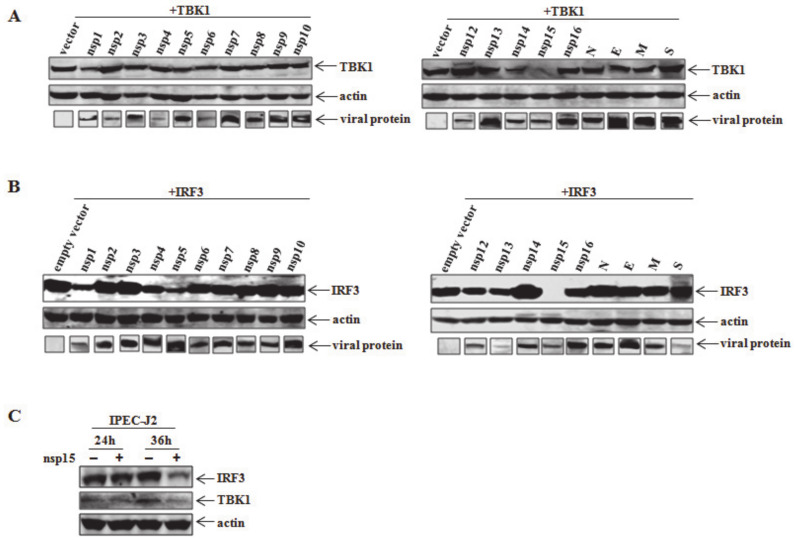
PEDV nsp15 reduces TBK1 and IRF3 expression at 36 h post transfection. (**A**) HEK293T cells were grown in 12-well plates and co-transfected with individual PEDV encoded protein (Flag tagged) and TBK1 (HA tagged). At 36 h posttransfection, cells were collected and determined by using anti-flag and HA antibodies. (**B**) HEK293T cells were seeded in 12-well plates followed by co-transfection with PEDV viral protein (Flag tagged) and IRF3 (HA tagged). At 36 h posttransfection, cells were subjected to western blot analysis with anti-flag and HA antibodies, respectively. (**C**) Cells were transfected with PEDV nsp15 (Flag tagged) for indicated culture, and then endogenous levels of TBK1 and IRF3 were determined by western blot using specific TBK1 or IRF3 antibody.

To explore which viral protein contributes to the reduction of TBK1 and IRF3, HEK293T cells were co-transfected with TBK1 or IRF3 and each PEDV encoded protein. At 36 h post transfection, cell samples were collected and lysed for detection of TBK1 or IRF3 expression. Several viral proteins were involved in the reduction of TBK1 or IRF3 expressions to varying extents, e.g., nsp1, nsp14, and nsp15 for TBK1 and nsp1, nsp4, nsp5, and nsp15 for IRF3, among which nsp15 can evidently downregulate the expression of either TBK1 or IRF3 compared with other viral proteins (Figure 2A,B). Moreover, endogenous TBK1 and IRF3 were also reduced followed by the ectopic overexpression of nsp15 at 36 h post transfection (Figure 2C). Therefore, we mainly focused on PEDV nsp15 as the research target in this study to investigate its role in modulating TBK1 and IRF3 expressions.

### 3.3. Involvement of EndoU Activity of PEDV nsp15 in TBK1 and IRF3 Reduction

**Figure 3:**
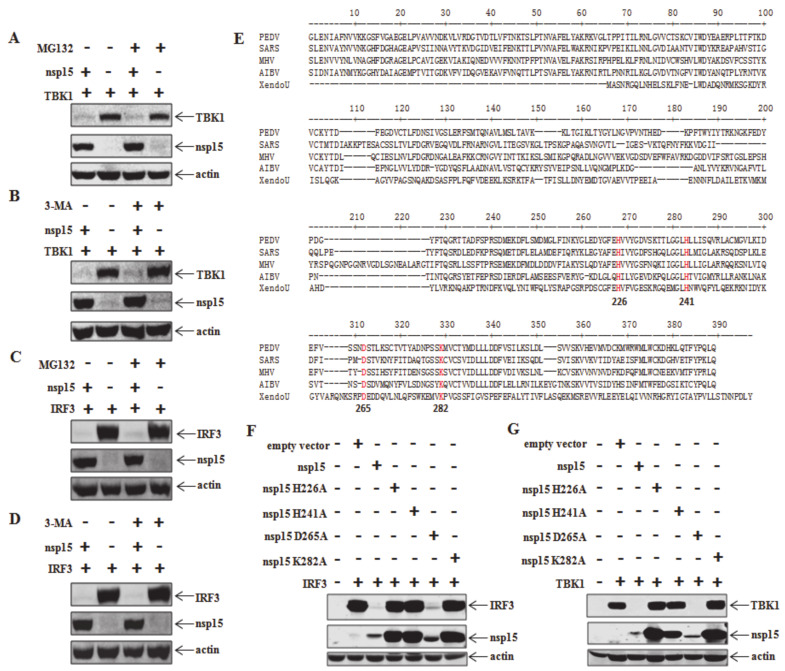
PEDV-induced reduction in TBK1 and IRF3 expression is due to PEDV nsp15 endoribonuclease activity, but not proteasome or autophagy-mediated mechanisms. (**A**,**C**) HEK293T cells were co-transfected with nsp15 (Flag tagged) and TBK1 or IRF3 (HA tagged) and were treated with the proteasome inhibitor MG132 (2 μM) or carrier control DMSO. Detergent lysates were collected and subjected to reducing SDS-PAGE and immunoblotting with anti-flag and HA antibodies. (**B**,**D**) HEK293T cells were co-transfected with nsp15 (Flag tagged) and TBK1 or IRF3 (HA tagged) and were treated with 3-MA (5 mM) for further culture. At 36 h post transfection, cell lysates were subjected to blotting with corresponding antibody. (**E**) Alignments of the nsp15 orthologs from several coronaviruses and *X. laevis* EndoU. The amino acid sequence of the PEDV nsp15 (PEDV, KT323979) was aligned with orthologs of severe acute respiratory syndrome coronavirus (SARS, KF514422), murine hepatitis virus (MHV, KF268339), avian infectious bronchitis virus (AIBV, NC_001451) and *Xenopus laevis* (XendoU, BC169902) using DNASTAR software. Gaps in the sequence alignment are denoted by hyphens. Residues with red are the conserved residues critical for nsp15 activity as previously described. The number below the red residue indicates its corresponding position at PEDV nsp15 protein, respectively. (**F**,**G**) Four mutants (H226A, H241A, D265A and K282A) of PEDV nsp15 were constructed by mutating the corresponding conserved residue mentioned in (**E**) into alanine using site-directed mutagenesis kit. HEK293T cells were then co-transfected with TBK1 or IRF3 and wild-type PEDV nsp15, the constructed nsp15 mutant (H226A, H241A, D265A or K282A) or empty vector. At 36 h post transfection, cell samples were subjected to immunoblotting with antibodies to flag, HA or β-actin (loading control).

Within eukaryotic cells, there are two major intracellular protein degradation pathways: the ubiquitin-proteasome system and autophagy [36]. The proteasomal degradation pathway has high selectivity and the proteasome generally recognizes ubiquitinated substrates [37]. By contrast, autophagy is a highly conserved process for degrading redundant cellular components by encircling them with membrane followed by a fusion of the vesicle with lysosomes [38]. Therefore, to determine the mechanism that might be responsible for the depletion of TBK1 and IRF3 by nsp15, the expression levels of TBK1 and IRF3 proteins were examined in cells treated with a protease inhibitor MG132 [39,40]. As shown in Figure 3A,C, treatment with MG132 cannot block the downregulation of TBK1 and IRF3 in HEK293T cells with co-transfection of TBK1 or IRF3 together with nsp15, thus not suggesting the proteasome-mediated degradation of TBK1 and IRF3 by nsp15. Additionally, we tested the possible role of autophagy in the reduction of TBK1 and IRF3 by treating cells with 3-MA, which is commonly used to inhibit autophagy [38,41]. We observed that 3-MA treatment did not inhibit TBK1 or IRF3 downregulation in HEK293T cells co-transfected with TBK1 or IRF3 along with nsp15 (Figure 3B,D). These data indicate that downregulation of TBK1 and IRF3 by nsp15 is not through the ubiquitin-proteasome system and autophagy.

Coronavirus nsp15 has been reported as a uridine-specific endoribonuclease and nuclease activities as well as crystal structure have been well identified as previously described [32,42,43,44,45]. Four inactive mutants of SARS-CoV nsp15, including H234A, H249A, D272A, and K289A, have been identified to lose the cleavage activity for substrate RNA due to loss of endoribonuclease activity, indicating that these four residues were critical for maintaining the nuclease activity of SARS-CoV nsp15 [42]. Based on amino acid sequence alignment of PEDV nsp15 with other coronavirus orthologs as well as XendoU from *X. laevis*, the four mentioned residues were also conserved in PEDV nsp15 and were denoted in red with the corresponding position in PEDV nsp15 sequence below (Figure 3E). Here, we asked whether TBK1 or IRF3 downregulation by PEDV nsp15 is dependent on its endoribonuclease activity. To determine whether the amino acid residues are also required for PEDV nsp15 endonuclease activity, four mutants (H226A, H241A, D265A, and K282A) were constructed by mutating corresponding residue of PEDV nsp15 to alanine. HEK293T cells were co-transfected with TBK1 or IRF3 together with nsp15 or constructed mutants following the detection of TBK1 and IRF3 by Western blot. It was demonstrated that nsp15 and D265A mutant can obviously reduce the expression levels of TBK1 and IRF3 post transfection. In contrast, the reduction of TBK1 or IRF3 expression was blocked when HEK293T cells were co-transfected with TBK1 or IRF3 and the remaining mutants (H226A, H241A, and K282A), indicating that residues of H^226^, H^241^, and K^282^ but not D^265^ are critical for the endoribonuclease activity of PEDV nsp15 (Figure 3F,G).

### 3.4. PEDV nsp15 Induces Reduction in RNA Levels of TBK1 and IRF3

**Figure 4:**
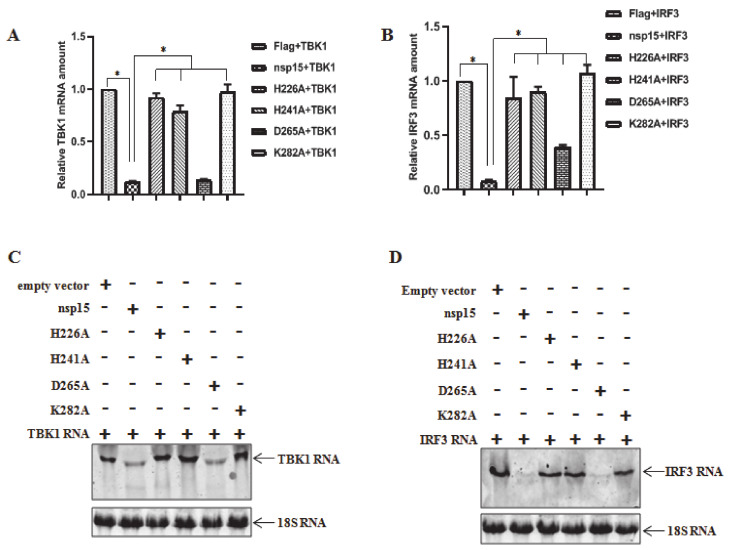
RNA reduction of TBK1 and IRF3 by PEDV nsp15 in an endoribonuclease activity dependent manner. (**A**,**B**) HEK293T cells were co-transfected with TBK1 or IRF3 and PEDV nsp15 expression plasmid (PEDV nsp15, H226A, H241A, D265A or K282A) or empty vector, followed by quantitative RT-PCR using the primers listed in Table 1. Three independent experiments were performed in triplicate, and values are the means ± SD for all three experiments. *, *p* < 0.05. (**C**,**D**) HEK293T cells were co-transfected with TBK1 or IRF3 and PEDV nsp15 expression plasmid (PEDV nsp15, H226A, H241A, D265A and K282A) or empty vector. At 36h post transfection, RNA were extracted from the collected cell samples followed by RNA detection by Northern blot assay using the designed specific probes (Table 1) as described in the Materials and methods.

Combined with the previous results, we hypothesized that PEDV nsp15 contributed to reduction of TBK1 and IRF3 expression by targeted mRNA level degradation in an EndoU activity dependent manner. To test this hypothesis, HEK293T cells were co-transfected with TBK1 or IRF3 and PEDV nsp15 as well as the constructed mutants followed by quantitative analysis of TBK1 or IRF3 mRNA level with the primers listed in Table 1. As shown in Figure 4A, the relative mRNA level of TBK1 was significantly more decreased in PEDV nsp15 and D265A transfected cells than in other mutants and empty vector transfected cells, which suggested that PEDV nsp15 can reduce TBK1 expression by downregulating the TBK1 mRNA levels dependent on its EndoU activity. Similar results were obtained that nsp15 can reduce IRF3 expression by decreasing the IRF3 mRNA levels in an EndoU dependent manner (Figure 4B). In addition, the mechanism was further verified by northern blot assay using the specific probe as designed in Table 1, following co-transfection with TBK1 or IRF3 and PEDV nsp15 as well as the constructed mutants in HEK293T cells. Consistent with the quantitative results by real time PCR assay, TBK1 or IRF3 RNA was evidently more reduced in nsp15 and D265A mutant transfected cells than in mutant H226A, H241A, K282A, or empty vector control transfected cells (Figure 4C,D). These data demonstrate that PEDV nsp15 can reduce TBK1 and IRF3 expression by the targeted degradation of TBK1 and IRF3 mRNA.

### 3.5. Direct Degradation of TBK1 and IRF3 mRNA by PEDV nsp15 In Vitro

**Figure 5:**
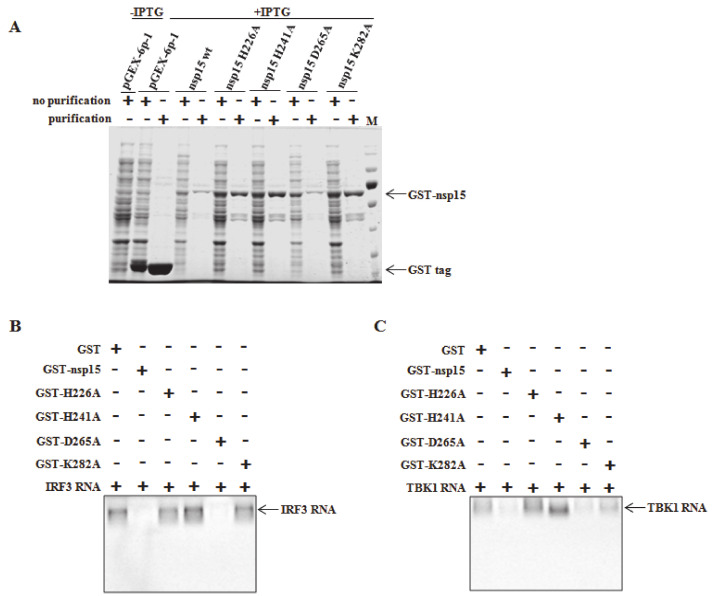
Degradation of TBK1 and IFR3 RNA by PEDV nsp15. (**A**) Wild-type PEDV nsp15 as well as its mutants (H226A, H241A, D265A and K282A) were cloned into prokaryotic expression vector of pGEX-6p-1, respectively. Targeted recombinant protein were expressed post induction with IPTG at the concentration of 0.5 mM and then purified by GST tag purification kit as the manufacture’s instruction (GE Healthcare Life Sciences). (**B**,**C**) Targeted RNA was transcribed and purified in vitro as described in the MATERIALS AND METHODS. endoribonuclease activity assay was performed by incubating the transcriptional RNA (TBK1 or IRF3) with purified recombinant PEDV nsp15 proteins (PEDV nsp15, H226A, H241A, D265A or K282A) or GST tag protein as a control at 37 °C for 1 h followed by RNA detection of TBK1 or IRF3 RNA as described in the Materials and methods.

We next investigated whether PEDV nsp15 can directly degrade mRNA of TBK1 and IRF3. To this end, wild-type and four mutant versions of PEDV nsp15 were produced as GST-tagged proteins and purified under mild conditions by the addition of reduced glutathione to the elution buffer as the manufacture’s instruction (GE Healthcare Life Sciences). As shown in Figure 5A, recombinant wild-type PEDV nsp15 and four mutants (H226A, H241A, D265A, and K282A) were purified successfully at the expected molecular weight of 66 kDa by SDS-PAGE analysis. The TBK1 and IRF3 sequences were amplified by PCR and subsequently cloned into the pGEM-T Easy vector containing a T7 RNA polymerase promoter upstream of the multiple cloning region to construct the recombinant plasmids of pGEM-T/TBK1 and pGEM-T/IRF3, respectively. In vitro-transcribed RNAs of TBK1 and IRF3 were synthesized from the constructed recombinant DNA templates (pGEM-T/TBK1 and pGEM-T/IRF3) by the RiboMAX™ large scale RNA production systems (Promega, USA). The GST-purified PEDV nsp15 proteins were incubated with the generated TBK1 and IRF3 mRNA as substrate in presence of Mn^2+^, a known cofactor for the endoribonuclease activity. [32,46]. It was revealed that synthesized TBK1 and IRF3 mRNA were effectively reduced post incubation with wild-type PEDV nsp15 and D265A mutant, but not with the other mutant derivatives (H226A, H241A, and K282A), demonstrating the PEDV nsp15 can directly degrade TBK1 and IRF3 mRNA dependent on its endoribonuclease activity (Figure 5B,C).

### 3.6. Suppression of TBK1 and IRF3 Mediated IFN Response by PEDV nsp15

**Figure 6:**
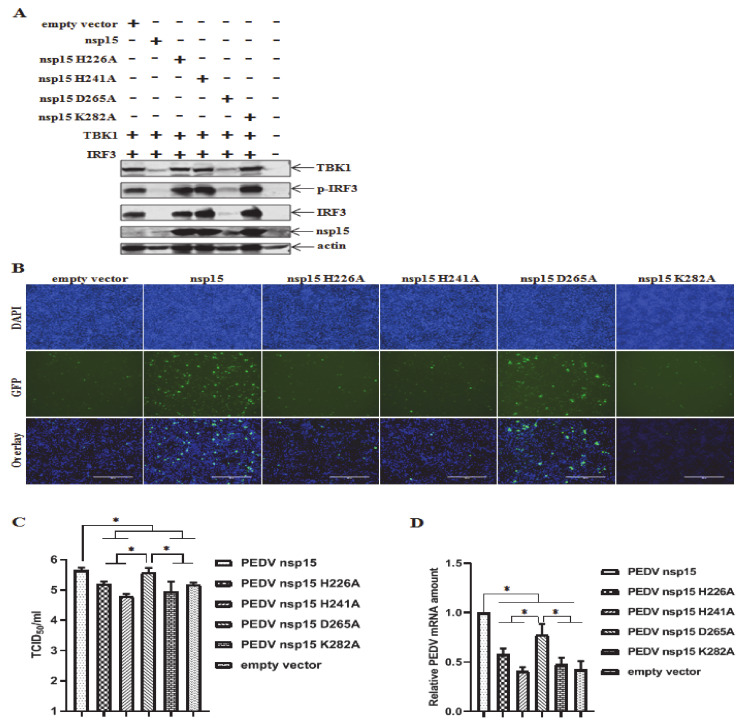
PEDV nsp15 inhibits IFN production dependent on its endoribonuclease activity. (**A**) HEK293T cells were co-transfected with TBK1 (HA tagged, 0.6 μg) and IRF3 (flag tagged, 0.6 μg) along with 0.6 μg of nsp15 expression plasmid (flag tagged, PEDV nsp15, H226A, H241A, D265A or K282A) or empty vector as a control. At 36 h post transfection, cell supernatants were collected for further use stated otherwise and cell samples were subjected to western blot analysis with antibodies against p-IRF3 (Ser396), flag, HA, or β-actin. (**B**) IPEC-J2 cells were cultured in 24-well plates for 24 h and then pretreated with the collected supernatant from experiment (**A**) with three-fold dilution for further 24 h, followed by VSV-GFP inoculation at MOI of 0.1. The VSV-GFP was visualized at 10 h post infection with an Olympus inverted fluorescence microscope equipped with a camera. The representative results were displayed by three different channels (transmitted, GFP, and overlay) for each treatment (100 ×). (**C**,**D**) Vero E6 and IPEC-J2 cells were seeded in 24-well plates and pretreated with collected supernatant as described in (**B**) prior to PEDV infection at MOI of 0.1 for Vero E6 (**C**) or 1.0 for IPEC-J2 cells (**D**). At 36 h post infection, the virus titer was determined by TCID_50_ or quantitative RT-PCR. Results represent the mean ± SD for three independent experiments. *, *p* < 0.05. The *p* value is calculated using Student’s *t*-test.

Type I IFNs are transcriptionally regulated, and are induced following recognition of pathogen components during infection. TBK1 and IRF3 are the key effectors during viral infections to induce IFN production [47]. Following stimulation with virus components including dsRNA, IRF3 becomes phosphorylated by the serine-threonine kinases TANK-binding kinase-1 (TBK1) or the inducible IκB kinase (IKK-i/IKKε) [48,49]. IRF-3 then dimerizes, translocates into the nucleus, and combines with the co-activator CBP/P300 to activate the expression of IFNβ [15]. To determine whether PEDV nsp15 modulates the phosphorylated IRF3, cells were co-transfected with TBK1 and IRF3 along with PEDV nsp15 as well as its mutant derivatives (H226A, H241A, D265A, and K282A) and then collected to examine the phosphorylated IRF3 levels by Western blot. As anticipated, the TBK1 stimulation led to the IRF3 phosphorylation in empty vector transfected cells. However, the IRF3 phosphorylations were significantly inhibited in nsp15 and D265A mutant transfected cells compared to the remaining mutants (H226A, H241A, and K282A) transfected cells, suggesting that PEDV nsp15 impeded IRF3 expression as well as IRF3 phosphorylation dependent on its EndoU activity (Figure 6A). Vesicular stomatitis virus (VSV) is frequently used for the assessment assay of IFN activity [50]. To determine the role of nsp15 in regulation of IFN production, HEK293T cells were co-transfected with TBK1 and IRF3 together with wild-type nsp15, individual nsp15 mutant or empty vector and cell supernatants were collected at 30 h post co-transfection for determining the status of IFN secretion. IPEC-J2 cells were treated with the supernatant followed by inoculation of VSV-GFP at MOI of 0.1. Fluorescence was visualized at 10 h post infection with an Olympus inverted fluorescence microscope equipped with a camera. VSV-GFP infection was evident in treatments with supernatant from nsp15 and D265A mutant transfected cells, and conversely VSV-GFP infection was obviously inhibited in treatments with supernatant from cells transfected with the remaining mutants (H226A, H241A, and K282A) or empty vector, demonstrating that nsp15 was an antagonistic protein in IFN production (Figure 6B). Moreover, we continued to investigate the effects of collected supernatant on PEDV infection in Vero E6 and IPEC-J2 cells. Vero E6 cells were treated with the individual collected supernatant as mentioned above prior to PEDV infection, cell samples were then collected and subjected to virus titration by TCID_50_ assay. PEDV infection was significantly enhanced in cells treated with supernatant from wild-type nsp15 and D265A mutant transfected cells than that from mutant H226A, H241A, K282A or empty vector transfected cells (Figure 6C), confirming that PEDV nsp15 can evade the host antiviral response by antagonizing IFN production. Meanwhile, a PEDV infection assay was further performed in IPEC-J2 cells, a target cell line for PEDV infection in vivo. Similar results were obtained in that PEDV nsp15 can facilitate PEDV infection, based on PEDV genomic quantitation by real-time PCR assay instead of by TCID_50_ assay due to its low susceptibility to IPEC-J2 cells (Figure 6D). These data collectively demonstrate that nsp15 can promote PEDV infection by limiting IFN secretion dependent on its endoribonuclease activity.

### 3.7. PEDV nsp15 Facilitates PEDV Replication by Disrupting the Antiviral Response of Type I Interferon

**Figure 7:**
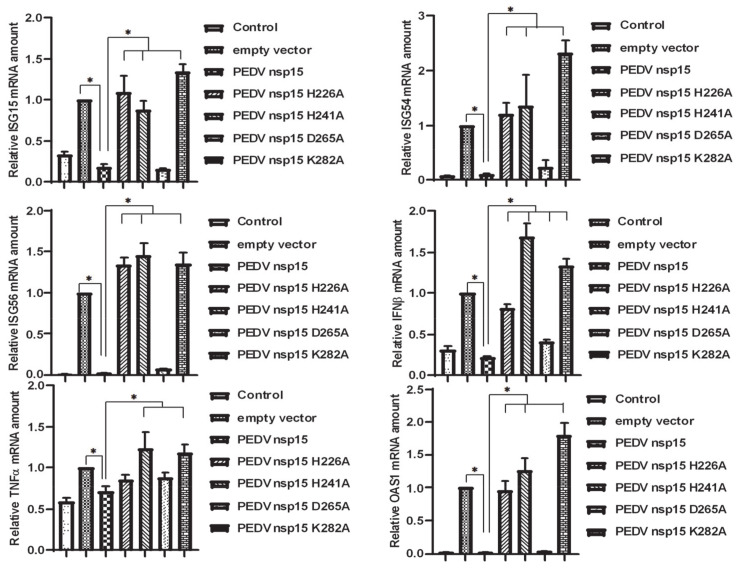
PEDV nsp15 negatively regulates TBK1 and IRF3 mediated induction of antiviral molecules. HEK293T cells were cells were co-transfected with TBK1 and IRF3 along with PEDV nsp15 expression plasmid (PEDV nsp15, H226A, H241A, D265A or K282A) or empty vector for 36 h. The RNA levels of innate immune related molecules, IFNβ, TNFα, ISG15, ISG54, ISG56 and OAS1, were determined by quantitative RT-PCR used the primers listed in Table 1. The results are representative of three independent experiments (mean ± SD). *, *p* < 0.05. The *p* value is calculated using Student’s *t*-test.

IFN-I is the key innate immune cytokine produced by cells to trigger antiviral function [51,52]. Therefore, we assessed the effect of nsp15 on the IFN mediated antiviral response signaling pathway. Here, HEK293 cells were co-transfected with TBK1 and IRF3 together with wild-type nsp15, nsp15 mutants, or empty vector control and cells samples were collected to investigate the effects of nsp15 on induction of innate antiviral molecules. Quantitative RT-PCR showed that mRNA levels of immune related molecules, such as IFNβ, TNFα, OAS1, ISG15, ISG54, and ISG56, were significantly disrupted by nsp15 and mutant D265A post transfection compared to that by empty vector control. However, the disruptions were impeded by the other nsp15 mutants that impaired the endoribonuclease activities (Figure 7), suggesting that PEDV nsp15 restrains cellular antiviral activity and thus facilitates PEDV infection.

## 4. Discussion

The host innate immune system is the first line of defense against virus invasion through production of IFNs as well as various other cytokines. Innate immune responses are activated through host pattern recognition receptors (PRRs), which recognize pathogen-associated molecular patterns [53]. IFNs exert antiviral effects through inducing the expression of hundreds of ISGs [52,54,55]. However, during coevolution with their host, viruses always evolve diverse strategies to escape and even inhibit host IFN responses [17,19,26,27,28,29,47,56].

PEDV has acquired multiple mechanisms that avoid the action of IFN by preventing the binding of viral products to cellular sensors. It was revealed that PEDV N protein antagonized IFN production by preventing TBK1 from interaction with IRF3 [27]. Of the several known viral evasion strategies, the cleavage of crucial innate immune molecules, including adaptors, kinases, and transcriptional factors, are considered to be a particularly powerful way for viruses to escape the innate immune response. For example, the 3C-like protease of PEDV and porcine delta coronavirus (PDCoV), disrupts type I IFN signaling by cleaving the NF-κB essential modulator (NEMO) [26,57]. In addition, PDCoV nsp5 antagonizes type I IFN signaling by cleaving STAT2, an essential factor for IFN responses [19]. Furthermore, the ubiquitination and deubiquitination are highly regulated post-translational modification processes in modulating the antiviral innate immune response. Within the cells, polyubiquitination plays several different roles depending upon the attachment position on the target proteins, and linked polyubiquitin chains regulate the proteasomal degradation of target proteins [25,58,59]. Multiple ubiquitin ligases and ubiquitin-binding scaffold proteins contribute to the positive regulation of the IFN response, such as RIG-I, TRAF2, TRAF6, and TBK1. Previous studies have indicated that PEDV PLP2 significantly inhibits the ubiquitination of RIG-I and STING, which is essential for the activation of type I IFN signaling [58]. Meanwhile, the proteasomal degradation of target proteins for the IFN response can also be achieved by viruses through the removal of K48 polyubiquitin chains [59,60,61]. PEDV-induced STAT1 degradation inhibits type I interferon signalling in a proteasome-dependent manner [25]. However, viruses are not just limited to the mentioned strategies to antagonize IFN responses. In this study, we first identified that PEDV nsp15 was capable of subverting the IFN response by the RNA degradation of TBK1 and IRF3, which differentiated from the strategies utilized by other coronavirus orthologues previously described.

**Figure 8:**
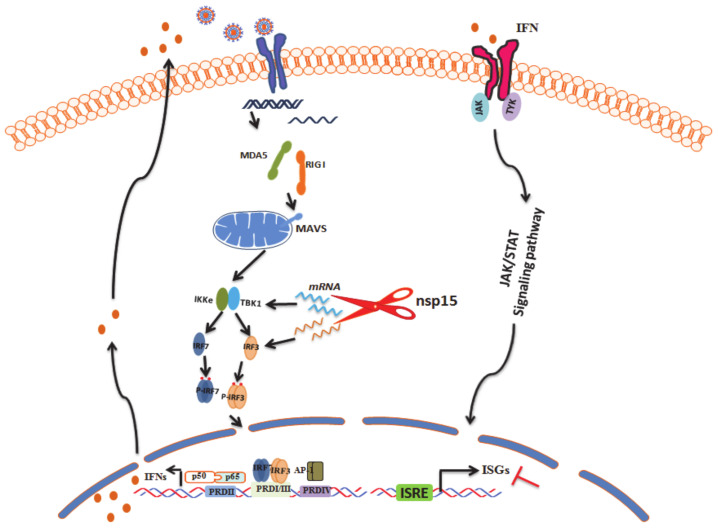
PEDV nsp15 antagonizes host antiviral response by degradating the RNA levels of TBK1 and IRF3. During PEDV infection, the RNA transcriptional levels of TBK1 and IRF3 were degraded by viral encoded nsp15 protein in an endoribonuclease activity dependent manner, resulting in reduction of endogenous TBK1 and IRF3. The decreased TBK1 and IRF3, two key effectors during IFN signaling pathway, circumvent IFN production and IFN mediated antiviral response.

The functions of nsp15 of coronaviruses (nsp11 in arteriviruses), an endoribonuclease encoded by nidoviruses, have received more attention. Previous studies showed that the nsp15 encoded by SARS-CoV [32], MHV [62,63], PEDV [45], PDCoV [64], and the nsp11 encoded by PRRSV [65,66] can antagonize antiviral innate immune responses by utilizing the different mechanisms involved, e.g., by mediating the evasion of viral dsRNA by host for MHV and HCoV-229E [62,63], by suppressing both MAVS and RIG-I expression for PRRSV [66], by impairing the activation of transcription factor NF-κB for PDCoV [64], or by inhibiting MAVS-induced apoptosis for SARS-CoV [67]. PEDV nsp15 of is a 339-residue polypeptide that results from the cleavage of pp1ab at sites ^6139^NLQ↓GLE^6144^ and ^6478^QLQ↓ASE^6483^ by the main protease nsp5. Several recent studies have focused on the structural and functional characterization of coronavirus nsp15 due to its potential importance as a drug target. It has been reported that the EndoU activity of PEDV nsp15 is not required for PEDV replication in Vero cells. However, the EndoU activity is involved in the suppression of host IFN response in epithelial cells and macrophages in vitro, and subsequently can facilitate pathogenesis development in vivo by enhancing viral replication and shedding [45]. Although previous studies have reported PEDV nsp15 as a key virulence factor that suppressed IFN responses in vitro and facilitated PEDV replication, the underlying mechanism remains unknown. In this study, we found that endogenous TBK1 and IRF3 were downregulated post PEDV by previous iTRAQ assay and Western blot analysis. Nsp15 was selected as the investigation candidate due to its evident effect on TBK1 and IRF3 reduction post co-transfection with each viral encoded protein. It was exhibited that PEDV nsp15 was capable of downregulating the expression of TBK1 and IRF3 proteins by the degradation of the RNA of TBK1 and IRF3 in an endoribonuclease activity dependent manner, while residues of H226A, H241A, and K282A were critical for the endoribonuclease activity of PEDV nsp15, but not D265A. The reason that results in these differences remains unclear. Whether these structure differences result in different mechanisms used to antagonize IFN production remains a subject of further study (Figure 8), elucidating a novel antagonistic mechanism utilized by PEDV to counter the antiviral response.

In summary, our data reveal that PEDV nsp15 acts as an IFN antagonist to inhibit immune response by the RNA level degradation of TBK1 and IRF3, key ingredients involved in the IFN signaling pathway dependent on its endoribonuclease activity, which will facilitate PEDV replication and the development of virus induced pathogenesis.

## Figures and Tables

**Table 1:** Real-time PCR primers used in this study.

Primer	Forward (5′→3′)	Reverse (5′→3′)	Usage
qIFNβ	CCATCTATGAGATGCTCCAG	TCCTTAGGATTTCCACTCTG	Quantitative RT-PCR
qTNFα	GCGTGGAGCTGAGAGATAAC	ATAGTCGGGCCGATTGATCT	Quantitative RT-PCR
qISG15	ATCACCCAGAAGATCGGCG	TCGAAGGTCAGCCAGAACAG	Quantitative RT-PCR
qISG54	CATTGACCCTCTGAGGCAAG	AGCGTGTCCTATTAGTTCC	Quantitative RT-PCR
qISG56	CATACATTTCCACTATGG	TACTCCAGGGCTTCATTCA	Quantitative RT-PCR
qOAS1	CTAGTCAAGCACTGGTACCA	ATCACAGGCCTGGGTTTCGT	Quantitative RT-PCR
qTBK1	TCTAATGCCTATGGACTACC	GCTCTCTCATACATATCAGG	Quantitative RT-PCR
qIRF3	ACCTGGAAGAGGAATTTCCG	CTGTCTTCGTGGGTATCAGA	Quantitative RT-PCR
TBK1-a	GGGGTACCCAGAGCACTTCTAATCATCTTTG	CCGCTCGAGCTAAAGACAGTCAACATTGCGAAG	pCAGGS/HA-TBK1
IRF3-a	GGAATTCGGAACTCAGAAGCCTCGGAT	GGGGTACCTCAATCCATGTCCTCCACCAGGT	pCAGGS/HA-IRF3
TBK1-t	CAGAGCACTTCTAATCATCTTTG	CTAAAGACAGTCAACATTGCGAAG	pGEM-T/TBK1
IRF3-t	ATGGGAACTCAGAAGCCTCGG	ATCCATGTCCTCCACCAGGTCC	pGEM-T/IRF3
H226A	ATTACGGCTTTGAGGCCGTTGTGTATGGTG	CACCATACACAACGGCCTCAAAGCCGTAAT	PEDV nsp15 H226A in pCAGGS
H241A	CCCTTGGTGGTTTAGCTCTACTAATTTCGC	GCGAAATTAGTAGAGCTAAACCACCAAGGG	PEDV nsp15 H241A in pCAGGS
D265A	TGTGTCTAGTAATGCTAGCACGTTAAAGTC	GACTTTAACGTGCTAGCATTACTAGACACA	PEDV nsp15 D265A in pCAGGS
H282A	ACAACCCTAGTAGTGCCATGGTTTGCACAT	ATGTGCAAACCATGGCACTACTAGGGTTGT	PEDV nsp15 H282A in pCAGGS
nsp15	GAATTCATGGGTCTTGAGAACATTGCTTTC	CTCGAGTCATTGAAGTTGTGGATAAAATGTC	nsp15 and mutants in pGEX-6p-1
TBK1-p	TTGAAGGGCCACGTAGGA	TGGTACTCAGAGGTTCCCG	Northern blot
IRF3-p	ACCTGGAAGAGGAATTTCCG	ACAGTCTGCTGGAAGACTTG	Northern blot
